# The efficacy of dopamine versus epinephrine for pediatric or neonatal septic shock: a meta-analysis of randomized controlled studies

**DOI:** 10.1186/s13052-019-0768-x

**Published:** 2020-01-14

**Authors:** Lingling Wen, Liangyin Xu

**Affiliations:** Department of Neonatology, Wenzhou People’s Hospital, The Wenzhou Third Clinical Institute Affiliated To Wenzhou Medical University, Wenzhou Maternal and Child Health Care Hospital, Wenzhou, 325000 Zhejiang China

**Keywords:** Dopamine, Epinephrine, Pediatric septic shock, Shock reversal, Randomized controlled trials

## Abstract

**Introduction:**

The efficacy of dopamine versus epinephrine for pediatric or neonatal septic shock remains controversial. We conduct a meta-analysis to explore the influence of dopamine versus epinephrine on shock reversal for pediatric or neonatal septic shock.

**Methods:**

We have searched PubMed, EMbase, Web of science, EBSCO, and Cochrane library databases through July 2019 for randomized controlled trials (RCTs) assessing the efficacy and safety of dopamine versus epinephrine for pediatric or neonatal septic shock.

**Results:**

Three RCTs are included in the meta-analysis. Overall for pediatric or neonatal septic shock, dopamine and epinephrine reveal comparable shock reversal within 1 h (risk ratios (RR) = 0.61; 95% CI = 0.16 to 2.31; *P* = 0.47), mortality (RR = 1.16; 95% CI = 0.87 to 1.55; *P* = 0.30), heart rate (standard mean differences (SMD) = 0.03; 95% CI = -0.28 to 0.34; *P* = 0.85), systolic blood pressure (SMD = -0.18; 95% CI = -0.69 to 0.33; *P* = 0.49), mean arterial pressure (SMD = -0.15; 95% CI = -1.64 to 1.34; *P* = 0.84) and adverse events (RR = 1.00; 95% CI = 0.94 to 1.07; *P* = 0.91).

**Conclusions:**

Dopamine and epinephrine show the comparable efficacy for the treatment of pediatric or neonatal septic shock.

## Introduction

Septic shock becomes the leading cause of mortality and morbidity among neonates and children worldwide [1–3]. Some studies report 10–50% of mortality in developed countries and up to 80% of mortality in developing countries [4–6]. The Surviving Sepsis Campaign 2012 guidelines have recommended dopamine as the first-line vasoactive agent in fluid-refractory septic shock [7]. Dopamine has a dose-dependent agonist effects on dopaminergic and adrenergic (α and β) receptors. Dopamine is inotropic via β-adrenergic stimulation in the dose range of 5–10 μg/kg/min, while it has both predominant inotropic effect and mild vasopressor effect via α1-adrenergic stimulation in the dosing range of 10–15 μg/kg/min. In the dose of more than 15 μg/kg/min, dopamine is predominantly a vasopressor (via α1-adrenergic effect) with minimal inotropic action [8].

Dopamine infusion in septic shock can reduce the release of prolactin, increase oxidative stress, suppress pro-inflammatory cytokine production and increase anti-inflammatory cytokine production [9, 10]. In young children and infants with decompensated hypotensive septic shock, dopamine response may be unpredictable because of receptor insensitivity to dopamine or catecholamine depletion [11]. In adults with septic shock, dopamine results in the increase in mortality and occurrence of arrhythmias when compared with norepinephrine [8, 12]. Epinephrine has the ability to increase mean arterial pressure and cardiac output, but may increase serum lactate and impair gut perfusion in septic shock [13, 14].

Recently, several studies have investigated the efficacy of dopamine versus epinephrine for pediatric or neonatal septic shock, but the results are conflicting [15–17]. This systematic review and meta-analysis of RCTs aims to assess the efficacy and safety of dopamine versus epinephrine for pediatric or neonatal septic shock.

## Materials and methods

This systematic review and meta-analysis are performed based on the guidance of the Preferred Reporting Items for Systematic Reviews and Meta-analysis statement and Cochrane Handbook for Systematic Reviews of Interventions [18, 19]. No ethical approval and patient consent are required because all analyses are based on previous published studies.

### Literature search and selection criteria

We have systematically searched several databases including PubMed, EMbase, Web of science, EBSCO, and the Cochrane library from inception to July 2019 with the following keywords: dopamine, and epinephrine, and septic shock, and pediatric or neonates. The inclusion criteria are as follows: (1) study design is RCT, (2) patients are diagnosed as pediatric or neonatal septic shock, and (3) intervention treatments are dopamine versus epinephrine.

### Data extraction and outcome measures

Some baseline information is extracted from the original studies, and they include first author, number of patients, age, the number of male, weight, mechanical ventilation requirement, and detail methods in two groups. Data are extracted independently by two investigators, and discrepancies are resolved by consensus. We have contacted the corresponding author to obtain the data when necessary.

The primary outcomes are shock reversal within 1 h and mortality. Secondary outcomes include heart rate, systolic blood pressure, mean arterial pressure and adverse events.

### Quality assessment in individual studies

The methodological quality of each RCT is assessed by the Jadad Scale which consists of three evaluation elements: randomization (0–2 points), blinding (0–2 points), dropouts and withdrawals (0–1 points) [20]. One point would be allocated to each element if they have been conducted and mentioned appropriately in the original article. The score of Jadad Scale varies from 0 to 5 points. An article with Jadad score ≤ 2 is considered to be of low quality. The study with Jadad score ≥ 3 is thought to be of high quality [21].

### Statistical analysis

We assess standard mean differences (SMD) with 95% confidence intervals (CIs) for continuous outcomes (heart rate, systolic blood pressure, and mean arterial pressure), and risk ratios (RR) with 95% CIs for dichotomous outcomes (shock reversal within 1 h, mortality, and adverse events). Heterogeneity is evaluated using the I^2^ statistic, and I^2^ > 50% indicates significant heterogeneity [22]. The random-effects model is used for all meta-analysis. We search for potential sources of heterogeneity for significant heterogeneity. Sensitivity analysis is performed to detect the influence of a single study on the overall estimate via omitting one study in turn or performing the subgroup analysis. Owing to the limited number (< 10) of included studies, publication bias is not assessed. Results are considered as statistically significant for *P* < 0.05. All statistical analyses are performed using Review Manager Version 5.3 (The Cochrane Collaboration, Software Update, Oxford, UK).

## Results

### Literature search, study characteristics and quality assessment

**Fig. 1:**
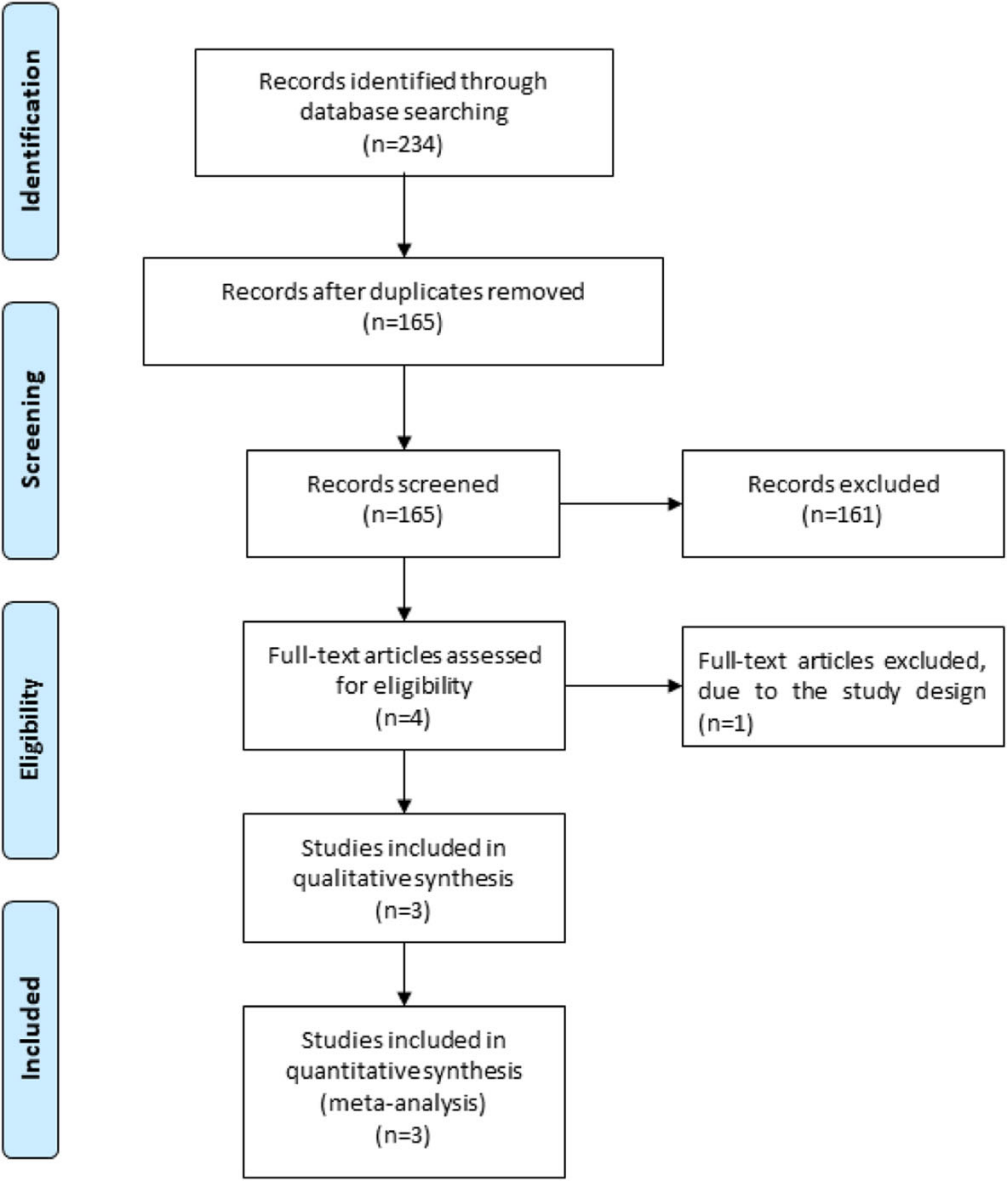
Flow diagram of study searching and selection process

Figure 1 shows the detail flowchart of the search and selection results. 234 potentially relevant articles are identified initially and three RCTs are finally included in the meta-analysis [15–17].

The baseline characteristics of three included RCTs are shown in Table 1. These studies are published between 2015 and 2018, and the total sample size is 220. The methods of dopamine or epinephrine are various in each RCT. Two studies involve pediatric septic shock [16, 17], and the remaining study involves neonatal septic shock [15].

**Table 1:** Characteristics of included studies

NO.	Author	Dopamine group						Epinephrine group						Jada scores
		Number	Age	Male (n)	Weight (g)	Mechanical ventilation requirement (n)	Methods	Number	Age	Male (n)	Weight (g)	Mechanical ventilation requirement (n)	Methods	
1	Baske 2018 [15]	20	–	13	1181 (892, 1540) g, median (interquartile range)	20	dopamine was initiated at 10 μg/kg/min, increased to 15μg/kg/min, thereafter to 20μg/kg/min (31–45 min) for neonatal septic shock	20	–	14	1100 (926, 1400) g	17	epinephrine was initiated at 0.2μg/kg/min, increased to 0.3 μg/kg/min, thereafter to 0.4 μg/kg/min (31–45 min).	4
2	Ramaswamy 2016 [16]	31	4 (0.8–8) years, median (interquartile range)	15	–	17	dopamine (in incremental doses, 10 to 15 to 20 μg/kg/min) till end points of resolution of shock for pediatric septic shock	29	7 (1–11) years	15	–	10	epinephrine (0.1 to 0.2 to 0.3 μg/kg/min) till end points of resolution of shock	4
3	Ventura 2015 [17]	63	39.6 ± 46.3 months	35	–	–	dopamine (5–10 μg/kg/min) through a peripheral or intraosseous line for pediatric septic shock	57	56.9 ± 58.2, months	35	–	–	epinephrine (0.1–0.3 μg/kg/min through a peripheral or intraosseous line	4

*BMI* body mass index

Two studies report shock reversal within 1 h and mortality [15, 16], two studies report heart rate, systolic blood pressure and mean arterial pressure [15, 17] and two studies report adverse events [16, 17]. Jadad scores of the three included studies are four, and all three studies have high-quality based on the quality assessment.

### Primary outcomes: shock reversal within 1 h and mortality

**Fig. 2:**
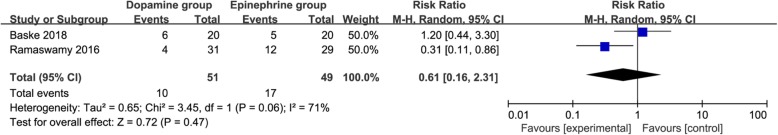
Forest plot for the meta-analysis of shock reversal within 1 h

**Fig. 3:**
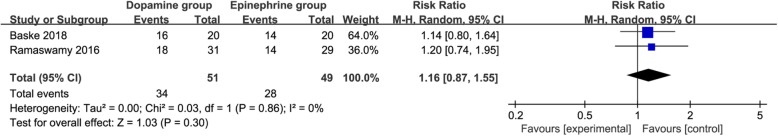
Forest plot for the meta-analysis of mortality

The random-effect model is used for the analysis of primary outcomes. The results find that dopamine and epinephrine intervention demonstrate comparable shock reversal within 1 h (RR = 0.61; 95% CI = 0.16 to 2.31; *P* = 0.47) with significant heterogeneity among the studies (I^2^ = 71%, heterogeneity *P* = 0.06, Fig. 2) and mortality (RR = 1.16; 95% CI = 0.87 to 1.55; *P* = 0.30) with no heterogeneity among the studies (I^2^ = 0%, heterogeneity *P* = 0.86, Fig. 3) for pediatric or neonatal septic shock.

### Sensitivity analysis

There is significant heterogeneity for shock reversal within 1 h, but no heterogeneity is observed for PFS for mortality. Because there are just two studies included for the analysis of shock reversal within 1 h, we do not perform the sensitivity analysis via omitting one study in turn.

### Secondary outcomes

**Fig. 4:**
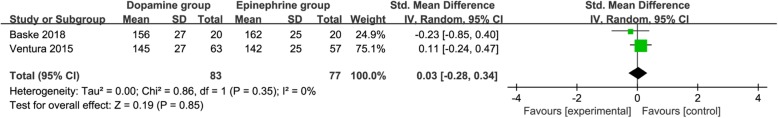
Forest plot for the meta-analysis of heart rate

**Fig. 5:**
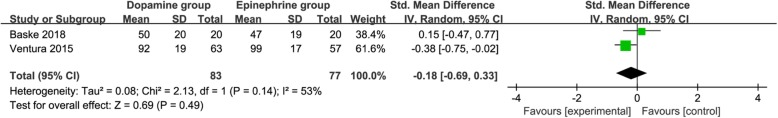
Forest plot for the meta-analysis of systolic blood pressure (mm Hg)

**Fig. 6:**
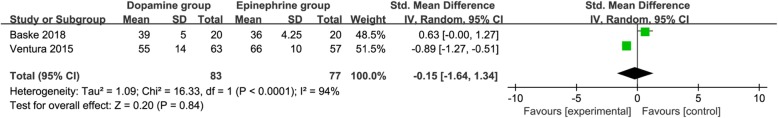
Forest plot for the meta-analysis of mean arterial pressure (mm Hg)

**Fig. 7:**
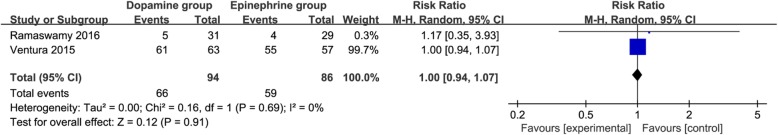
Forest plot for the meta-analysis of adverse events

In comparison with epinephrine intervention for pediatric or neonatal septic shock, dopamine shows similar heart rate (SMD = 0.03; 95% CI = -0.28 to 0.34; *P* = 0.85; Fig. 4), systolic blood pressure (SMD = -0.18; 95% CI = -0.69 to 0.33; *P* = 0.49; Fig. 5), mean arterial pressure (SMD = -0.15; 95% CI = -1.64 to 1.34; *P* = 0.84; Fig. 6) and adverse events (RR = 1.00; 95% CI = 0.94 to 1.07; *P* = 0.91; Fig. 7).

## Discussion

Both dopamine and epinephrine can provide vasopressor and inotropic actions [23–25]. Vasopressors serve as the first-line vasoactive drugs in the management of neonatal septic shock because of decreased systemic vascular resistance [26, 27]. Dopamine is recommended to be the first-line vasoactive agent in fluid-refractory septic shock [7]. It is also the first-line vasoactive drug in neonatal septic shock mainly through the release of norepinephrine from presynaptic vesicles [28–30]. Dopamine may be ineffective in sick neonates due to the depletion of norepinephrine stores within few hours of sickness onset [31].

In contrast, epinephrine acts directly on adrenergic receptors [23], and has the ability to decrease myocardial oxygen extraction ratio and increase the coronary sinus oxygen content in animal models [32]. Epinephrine is found to show three times more likely to achieve the resolution of shock within first hour of resuscitation than dopamine in pediatric fluid-refractory hypotensive septic shock. Early resolution of shock with epinephrine benefits to improve organ functions [16]. Our meta-analysis suggests that dopamine and epinephrine obtains the comparable shock reversal for pediatric or neonatal septic shock.

In adults with septic shock, strong evidence is observed that dopamine increases the mortality and adverse events [8, 12]. In another study, the mortality in children receiving dopamine is significantly increased than those taking epinephrine in the short period of time in pediatric septic shock [17]. However, there is no statistical difference of mortality between dopamine and epinephrine in the management of pediatric or neonatal septic shock based on this meta-analysis. In addition, no significance of heart rate, systolic blood pressure, mean arterial pressure or adverse events is observed between these two groups. Regarding the sensitivity analysis, significant heterogeneity is observed for shock reversal within 1 h (I^2^ = 71%, heterogeneity *P* = 0.06, Fig. 2), systolic blood pressure (I^2^ = 53%, heterogeneity *P* = 0.14, Fig. 5) and mean arterial pressure (I^2^ = 94%, heterogeneity *P* < 0.0001, Fig. 6). Many factors such as different population with septic shock, doses, duration and methods of drug use may result in this heterogeneity.

Several limitations exist in this meta-analysis. Firstly, our analysis is based on only three RCTs, and more RCTs with large sample size should be conducted to explore this issue. Next, there is significant heterogeneity, which may be caused by different population with septic shock, doses, duration and methods of drug use etc. Finally, it is not feasible to perform the subgroup analysis based on pediatric or neonatal septic shock based on limited RCTs.

## Conclusion

Dopamine and epinephrine shows the similar efficacy and safety for pediatric or neonatal septic shock, and more studies should be conducted to investigate this issue.

## Data Availability

Not applicable.

## Abbreviations

RCT: Randomized controlled trial
RRs: Risk ratios
Std. MD: Standard mean difference
